# A New Mathematical Framework for CMOS Si Photomultiplier Detection Rates in Quantum Cryptography

**DOI:** 10.3390/s26041386

**Published:** 2026-02-22

**Authors:** Tal Gofman, Yael Nemirovsky

**Affiliations:** Faculty of Electrical and Computer Engineering, Technion–Israel Institute of Technology, Haifa 3200003, Israel; talgofman@campus.technion.ac.il

**Keywords:** Discrete Variable Quantum Key Distribution (DV-QKD), Single-Photon Avalanche Diode (SPAD), Silicon Photomultiplier (SiPM), analog and digital SiPM, CMOS SPAD dead time, gigahertz quantum communication

## Abstract

The deployment of Discrete Variable Quantum Key Distribution (DV-QKD) in high-traffic, short-reach environments, such as intra-data center networks, is currently constrained by the saturation of single-photon detectors. While CMOS Single-Photon Avalanche Diodes (SPADs) offer a cost-effective solution, their Secure Key Rate (SKR) is limited by detector dead time. To the best of the authors’ knowledge, this work is the first to derive a generalized detection rate model for SiPMs that addresses the dead-time bottlenecks of gigahertz-rate quantum cryptography. While methods for managing deadtime via active optical switching have been proposed, our model quantifies the benefits of passive spatial multiplexing inherent in standard SiPM arrays. Furthermore, contrasting with models designed to optimize energy resolution or characterize nonlinear charge response to light pulses, our work focuses on maximizing the detection count rate. We derive exact detection rate models for both analog (paralyzable) and digital (non-paralyzable) SiPM architectures, incorporating correlated noise sources such as optical crosstalk and afterpulsing. Simulation results indicate that SiPMs can increase detection rates by over an order of magnitude compared to single SPADs.

## 1. Introduction

As quantum computing threatens classical cryptographic protocols, the integration of quantum secure methods to share encryption keys like Discrete Variable Quantum Key Distribution (DV-QKD) into commercial infrastructure becomes critical [1]. A particularly challenging environment is the intra-data center network, characterized by short-reach links (<10 km) and requirements for high-throughput key generation [2,3,4].

In these high-flux environments, the primary bottleneck for the quantum key generation rate is the dead time of the Single-Photon Detector (SPD) being used. While Superconducting Nanowire Single-Photon Detectors (SNSPDs) offer excellent performance, their cryogenic cooling requirements render them economically unviable for widespread data center deployment.

CMOS-based Silicon Single-Photon Avalanche Diodes (SPADs) operate at room temperature and are cost-effective but suffer from dead times (typically nanoseconds) that saturate detection rates well below the gigahertz regime required for modern data traffic [5,6,7].

This paper proposes the use of Silicon Photomultiplier (SiPM), an array of SPADs, to mitigate these limitations. By treating the SiPM not merely as an imaging device [8,9] but as a high-rate multi-pixel detector, we model how spatial multiplexing allows the detector to remain active even when individual pixels are dead, significantly extending the achievable key generation rate. To properly contextualize this approach, it is necessary to review the current landscape of detector modeling. State-of-the-art modeling of the SiPM response has largely focused on characterizing intrinsic device physics and optimizing energy resolution for spectroscopic applications, rather than maximizing digital count rates for communications. Extensive work has been done to describe the statistical nature of correlated noise. For instance, Schioppa derived exact recursive solutions to extract dark count and crosstalk rates from dark noise spectra [10], while Kawata et al. and Para rigorously characterized the geometric probability distribution of afterpulsing and its impact on excess noise factors [11,12]. Regarding saturation and non-linearity, recent models have incorporated the complex analog recovery of pixels. Rosado developed a statistical model describing the mean output charge and current linearity [13], accounting for the recovery of gain and photodetection efficiency described by Gallina et al. [14]. Similarly, Vinogradov et al. analyzed SiPM efficiency in terms of Photon Number Resolution (PNR) and Detective Quantum Efficiency (DQE) [15]. However, these studies primarily address the fidelity of pulse height measurements for calorimetry and medical imaging, rather than the maximum achievable photon counting throughput required for QKD. In the pursuit of overcoming this throughput bottleneck, previous studies have explored active dead-time management strategies. Notably, Polyakov et al. [16] demonstrated an ‘intelligent’ detection system that utilizes a high-speed 1-by-N optical switch to actively route incoming photons to available ‘live’ detectors based on their firing history. While this technique effectively minimizes the aggregate dead time, it requires real-time feedback logic to monitor detector states and actively steer photon paths. In contrast, our work focuses on passive spatial multiplexing inherent to standard SiPM. Unlike active switching, the SiPM allows the detector to remain responsive through the probabilistic distribution of photon arrivals across multiple pixels. This passive architecture eliminates the need for external routing logic and preserves the intrinsic statistical nature of the photon flux, a critical feature for the security of high-speed DV-QKD implementations. Section 2 reviews CMOS SPAD detection models, as reported in the literature.

Section 3 summarizes the innovation of this study: a generalization of the single SPAD detection models to the case of square SiPMs.

Section 4 validates the theoretical generalized model with a simulation showcasing the performance advantage of SiPMs over a single SPAD in high-rate single-photon detection applications like intra-data center DV-QKD. We also briefly address the effects of different combining logic architectures (OR/XOR) on saturation rates as analyzed by Gnecchi et al. [17], distinguishing between logic-limited and pixel-limited saturation regimes.

## 2. CMOS SPAD Detection Models

To model the SiPM, we first establish the behavior of its fundamental unit: the single SPAD. The detection rate is governed by the quenching mechanism, which dictates how the device resets after an avalanche.

SPADs employing active quenching circuits (AQCs) are typically modeled as non-paralyzable [18]. In this mode, the detector is insensitive during the dead time, $t_{d}$, but events occurring during this period do not extend the dead time. The detection rate, $R_{np}$, is given by:

(1) $$R_{np}=\frac{\lambda_{total}}{1+\lambda_{total}t_{d}}$$

where $\lambda_{total}$ is the total rate of avalanche triggering events. For a single SPAD, it includes avalanches due to photon absorption, dark count and afterpulsing (AP) events:

(2) $$\lambda_{total}=\lambda_{signal}+DCR+\lambda_{ap}$$

(3) $$\lambda_{signal}=\lambda_{ph}\cdot PDP\cdot FF$$

(4) $$\lambda_{ap}=\left(\lambda_{signal}+DCR\right)\frac{p_{ap}}{1-p_{ap}}$$

where DCR is the dark count rate, $\lambda_{signal},\lambda_{ap}$ are the avalanche rates due to photon absorption and AP, respectively, $\lambda_{ph}$ is the rate photons arrive at the detector, PDP is the photon detection probability, FF is the detector’s fill factor and $P_{ap}$ is the afterpulsing probability. Since AP arises from carrier trapping that can trigger subsequent events in a cascading manner, a single primary avalanche may initiate a correlated pulse train. Therefore, rather than relying on the single-event probability $P_{ap}$, we define the rate of AP-induced avalanches using the expectation value $\alpha =\frac{p_{ap}}{1-p_{ap}}$. This formulation models the geometric distribution of the afterpulsing chain established by Kawata et al. [11], and accounts for the cumulative increase in the effective count rate analyzed by Para [12].

Conversely, in high-flux regimes SPADs using passive quenching circuits (PQCs) are often paralyzable through the mechanism of dead time extension, which is described and demonstrated in [18,19]. Avalanches occurring during the recharge phase, while the applied voltage on the SPAD is below the readout’s sensing threshold, are not recorded but still discharge the capacitance, resetting the dead time. This leads to a drop in detection rate, $R_{p}$, at high fluxes:

(5) $$R_{p}=\lambda_{total}e^{-\lambda_{total}t_{d}}$$

**Figure 1:**
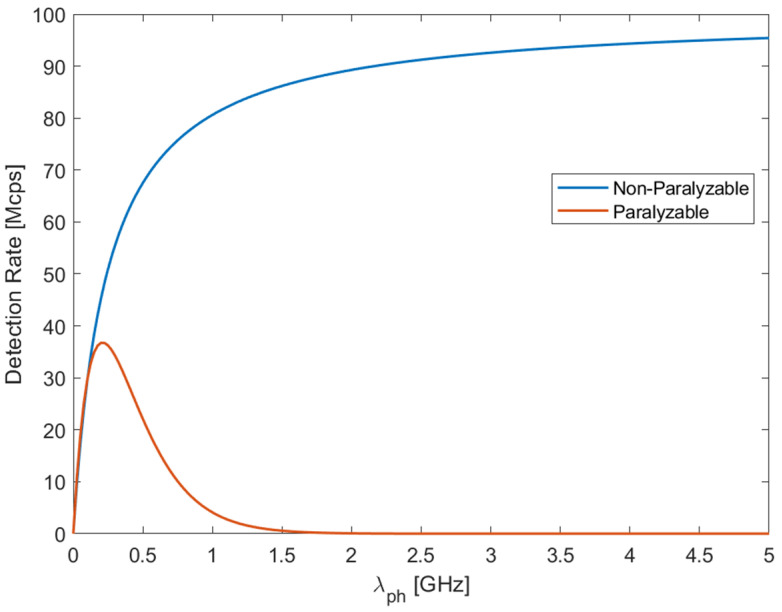
Detection rate models of paralyzable and non-paralyzable single SPAD as a function of the rate at which single photons arrive at the detector, $\lambda_{ph}$. For both models the SPAD parameters are $t_{d}=10\;ns\left(\frac{1}{t_{d}}=100\;MHz\right),FF=70\%,PDP=60\%,DCR=1\;kcps,P_{ap}=5\%$.

Single SPADs, regardless of the model, saturate at $\frac{1}{t_{d}}$ (non-paralyzable) and $\frac{1}{et_{d}}$ (paralyzable, e being Euler’s number) [18], effectively capping the detection rate (Figure 1).

In the following section, we generalize the detection rate formalism from Equations (1)–(5) to model SiPM detection rates (see Appendix A for the complete mathematical derivation).

## 3. Generalized Silicon Photomultiplier Detection Model

**Figure 2:**
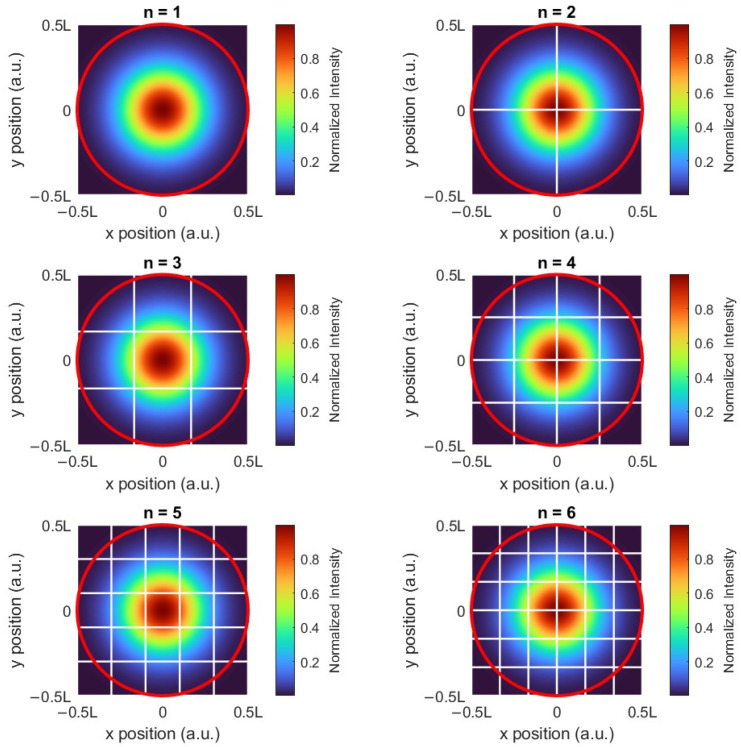
A Gaussian PDF spread on the SiPM with $N=n^{2}$ pixels (marked by the white lines), where 1≤n≤6. The probability that a photon will interact with the (i,j) pixel, $P_{i,j}$, is derived by integrating the PDF over the (i,j) pixel’s area. For all configurations the total SiPM area is $L^{2}$; i.e., as the number of array pixels increases, each pixel has a smaller area. To confine 99% of the PDF in the detector’s area (red circle), the side’s length is $L=3w\left(z\right)$, where w(z) is the Gaussian beam’s waist.

The simplest SiPM is a square nn array of SPADs (n-number of pixels in a row or column). To determine the effective detection rate of the array, we must account for the non-uniform photon distribution, assuming a Gaussian beam profile, representing the incident photon’s spatial probability density function (PDF). The rate of signal photons impinging on the (i,j) pixel is $\lambda_{signal_{i,j}}=\lambda_{signal}\cdot P_{i,j}$, where $P_{i,j}$ is the probability that a photon will impinge on the (i,j) pixel, derived from spatial integration of the Gaussian profile for pixel (i,j). Figure 2 illustrates the PDF distribution across individual SiPM pixels (SPADs). The primary avalanche rate for a pixel includes both avalanches due to photon absorption and dark count avalanches:

(6) $$\lambda_{primary_{i,j}}=\lambda_{signal_{i,j}}+DCR$$

**Figure 3:**
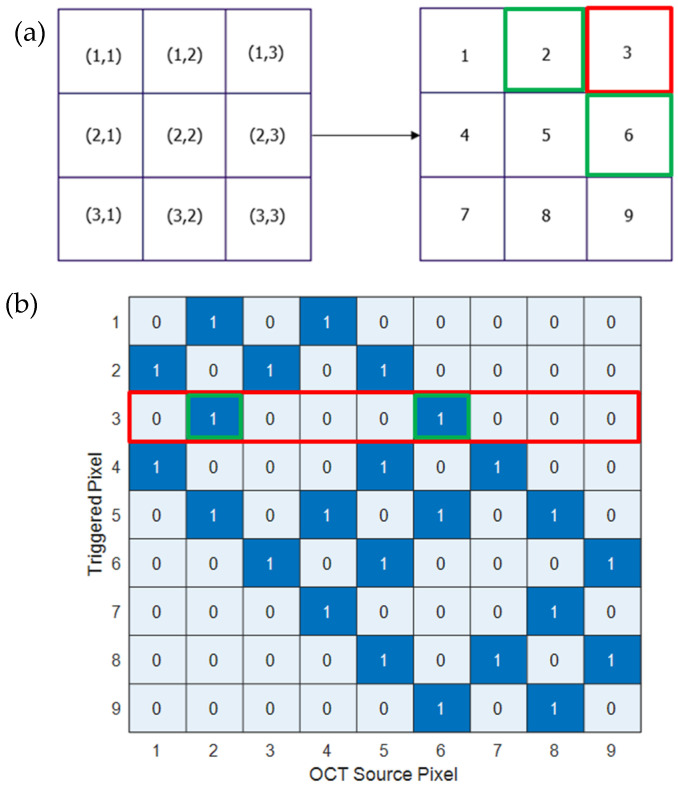
(**a**) Visual illustration of mapping from two-dimensional representation to one dimensional presentation for a 3 × 3 SiPM. (**b**) An $A_{9\;\times \;9}$ coupling matrix showing the relations between triggered pixels (rows) and the OCT source pixels (columns) according to the four nearest neighbors model [20], where the triggered pixel’s nearest neighbors (OCT source pixels) are noted by “1” (dark blue) and the rest noted by “0” (light blue). For example: pixel no. 3 on (**a**) is represented by the third row in the coupling matrix (red rectangle). Pixels no. 2 and 6 might trigger an OCT avalanche in pixel 3 as its nearest neighbors. They correspond to the second and sixth columns of the coupling matrix (green squares).

A major challenge in SiPM modeling is the cascading effect of correlated noise. An AP event could cascade into a series of AP avalanches in the same pixel, while an optical crosstalk (OCT) event could spatially cascade, triggering OCT avalanches in surrounding pixels. Furthermore, every AP event could trigger OCT avalanches in its neighbors and vice versa—a pixel triggered due to OCT could be triggered by AP afterwards. Thus, three coupled equations are required to derive the total avalanche rate of a single pixel in a SiPM:

(7) $$\lambda_{{xt}_{i,j}}=\left[\left(\sum_{\begin{array}{c} k=i-1\\ k\neq i \end{array}}^{i+1}\lambda_{{signal}_{k,j}}+{\lambda_{ap}}_{k,j}+\sum_{\begin{array}{c} l=j-1\\ l\neq j \end{array}}^{j+1}\lambda_{{signal}_{i,l}}+{\lambda_{ap}}_{i,l}\right)+4DCR\right]P_{xt}$$

(8) $$\lambda_{{ap}_{i,j}}=\left(\lambda_{{primary}_{i,j}}+\lambda_{{xt}_{i,j}}\right)\frac{P_{ap}}{1-P_{ap}}$$

(9) $$\lambda_{{total}_{i,j}}=\lambda_{{primary}_{i,j}}+\lambda_{{ap}_{i,j}}+\lambda_{{xt}_{i,j}}$$

where $\lambda_{total_{i,j}},\lambda_{ap_{i,j}}$ and $\lambda_{xt_{i,j}}$ are the (i,j) pixel’s total avalanche rate and avalanche rates due to AP and OCT events, respectively, and $P_{xt}$ is the probability that a pixel will be triggered by an OCT event from a neighboring pixel, according to the four nearest neighbors model derived in [20], and was found to agree with the experimental evidence presented in it. Substituting (8) in (7) gives:

(10) $$\lambda_{{xt}_{i,j}}=\left[\sum_{\begin{array}{c} k=i-1\\ k\neq i \end{array}}^{i+1}\left(\lambda_{{primary}_{k,j}}+\alpha \left(\lambda_{{primary}_{k,j}}+\lambda_{{xt}_{k,j}}\right)\right)+\sum_{\begin{array}{c} l=j-1\\ l\neq j \end{array}}^{j+1}\left(\lambda_{{primary}_{i,l}}+\alpha \left(\lambda_{{primary}_{i,l}}+\lambda_{{xt}_{i,l}}\right)\right)\right]P_{xt}$$

where $\alpha =\frac{P_{ap}}{1-P_{ap}}$. Defining N×1 column vectors where each element corresponds to the avalanche rate of a specific pixel in the array for $\lambda_{primary}$ and $\lambda_{xt}$ simplify the calculation and (10) becomes:

(11) $$\Lambda_{xt}={\left(I-P_{xt}\alpha A\right)}^{-1}AP_{xt}\left(1+\alpha \right)\Lambda_{primary}$$

where I is the identity matrix, and $A_{NxN}$ is a coupling matrix, representing how the $m_{th}$ element of $\Lambda_{xt}$ (corresponds to pixel (i,j) in the two-dimensional SiPM representation) might be triggered by OCT coming from avalanches in its nearest neighbors (as illustrated in Figure 3) (Of course, the possible r values are also determined by the triggered pixel location in the array. For example-when m=1, r cannot be r=m−1=0, as there is no such pixel in the array. Another example: for m=4, $A_{q,r}=0$ as r=3 means that pixel number 3 (Figure 3a) sourcing OCT, which could trigger pixel number 4, which cannot happen according to the four nearest neighbors model [20]):

$$A_{q,r}=\left\{\begin{array}{c} 1,\\ 1,\\ \begin{array}{c} 1,\\ 1,\\ 0, \end{array} \end{array}\begin{array}{c} q=m,r=m+n\\ q=m,r=m-n\\ \begin{array}{c} q=m,r=m+1\\ \begin{array}{c} q=m,r=m-1\\ otherwise \end{array} \end{array} \end{array}\right.$$

where 1≤m≤N, q marks the row corresponding to the OCT triggered pixel—m, and r marks the column representing the OCT source pixel. $A_{q,r}=1$ means pixel m could be triggered by OCT sourcing from its adjacent pixels, and $A_{q,r}=0$ means the pixel does not trigger an OCT to itself and non-adjacent pixels do not trigger OCT in pixel m as well.

Thus, the total avalanche rate can be described in a vector notation as a function of $\Lambda_{primary}$ and the correlated noise probabilities:

(12) $$\Lambda_{total}=\left(1+\frac{P_{ap}}{1-P_{ap}}\right)\left[I+P_{xt}\left(1+\frac{P_{ap}}{1-P_{ap}}\right){\left(I-P_{xt}\frac{P_{ap}}{1-P_{ap}}A\right)}^{-1}A\right]\Lambda_{primary}$$

The effective detection rate of the SiPM is the sum of the rates of individual pixels, processed according to their quenching architecture. For a Digital SiPM (dSiPM), where each pixel is actively quenched (non-paralyzable):

(13) $$R_{dSiPM}=\sum_{m=1}^{N}\frac{{\Lambda_{total}}_{m}}{1+\Lambda_{{total}_{m}}t_{d}}$$

For an Analog SiPM (aSiPM), where pixels are passively quenched (paralyzable):

(14) $$R_{aSiPM}=\sum_{m=1}^{N}\Lambda_{{total}_{m}}e^{-\Lambda_{{total}_{m}}t_{d}}$$

## 4. Simulation Results and Discussion

**Figure 4:**
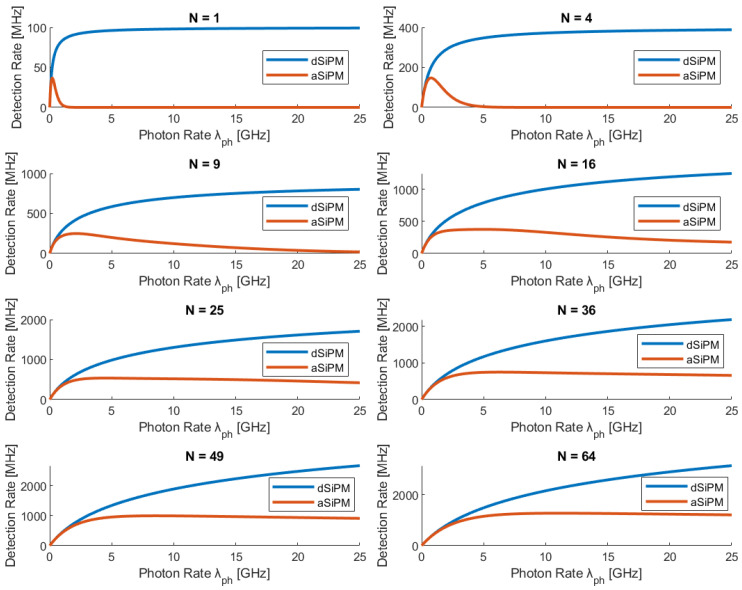
Detection rate of dSiPM and aSiPM for varying number of array pixels as a function of the rate at which single photons arrive at the detector, $\lambda_{ph}$. Array pixel parameters—$DCR=1\;kcps,P_{ap}=5\%,\varepsilon =10\%,FF=70\%,PDP=60\%$, ε is the probability that an OCT event will occur for the entire detector [20], usually determined empirically. As the number of array pixels increases, dSiPM saturates later, while the aSiPM becomes tolerant to larger values of $\lambda_{ph}$-instead of a narrow peak, and the maximum detection rate elongates into pseudo-saturation, delaying the aSiPM’s paralysis stage.

Simulations were conducted for SiPMs with varying pixel counts $\left(1\leq N\leq 64\right)$ under a Gaussian illumination profile (see Figure 4). Increasing N significantly delays saturation. For a SiPM constructed out of N≥16 SPADs the achievable detection rate is larger by more than an order of magnitude for both dSiPM and aSiPM. Also, the aSiPM demonstrates paralysis resistance; while individual pixels in an aSiPM are paralyzable, the array exhibits “pseudo-saturation”. As shown in the simulation results, for N≤9, the aSiPM detection rate matches the dSiPM up to a photon incident rate of $1\frac{Gph}{s}$. Beyond this, the dSiPM outperforms due to its non-paralyzable nature, but the aSiPM remains viable for rates far exceeding a single SPAD. Figure 4 clearly demonstrates the substantial benefits of employing SiPMs for short-reach, high-rate quantum communication applications such as DV-QKD in data-center environments.

While the presented model focuses on the statistical availability of pixels (passive spatial multiplexing), practical implementations of dSiPMs must also consider the bandwidth limitations imposed by the electronic readout architecture. As analyzed by Gnecchi et al. [17], the method of combining pixel outputs, typically via OR-trees or XOR-trees, introduces a secondary saturation mechanism known as “routing pile-up.” In an OR-tree architecture, coincident events triggered by multiple photons (or simultaneous signal and noise events) are merged into a single digital pulse if they overlap in time, potentially undercounting the true flux.

However, we determined that, for the specific constraints of intra-data center DV-QKD applications, which favor compact detectors, the choice of electronic architecture has a negligible impact on the saturation curves presented in Figure 4. According to the analysis in [17], particularly the relationship between array size and maximum count rate, the performance advantage of XOR-logic over OR-logic becomes significant only when the number of pixels is large enough (N>100) for the array’s effective dead time to approach the logic circuit’s minimum pulse width limits. For the regime modeled in this work (N≤64), the detection rate is dominated by the fundamental pixel dead time scaling rather than the combining logic’s saturation.

It is important to note that, while the merging of coincident events in OR-trees does not significantly alter the macroscopic throughput in this regime, it does have implications for the Quantum Bit Error Rate (QBER). Specifically, if an afterpulsing event and a simultaneous optical crosstalk event are merged due to routing pile-up, they are registered as a single false count, which obscures the true noise flux and affects the QBER differently than if each correlated noise event were resolved. A full analysis of how these specific coincidence mechanisms degrade the QBER requires a distinct probabilistic framework that goes beyond the throughput saturation model presented here.

## 5. Conclusions

This study is, to our knowledge, the first to generalize the detection rate models of single SPADs to SiPMs, SPAD arrays, explicitly accounting for cascading correlated noise sources. We showed that SiPMs overcome the dead-time limitations of single SPADs, enabling gigahertz-range detection rates essential for intra-data center DV-QKD.

## Data Availability

Data is available upon reasonable request from the correspondence author.

## Appendix A

In the case of SiPM, Equation (3) should be altered to describe the (i,j) pixel’s avalanche rate dependency on the probability that a photon will impinge it.

(A1) $$\lambda_{signal_{i,j}}=\lambda_{ph}\cdot FF\cdot PDP\cdot P_{i,j}$$

Coming to derive a model of the registered detection rate of a SiPM, $R_{SiPM}$, correlated and uncorrelated noise and corresponsive cascading phenomena should be taken into consideration. Under the assumption that all array pixels are identical, the DCR is also identical for all the pixels. A primary avalanche is defined as the result of either a photon being absorbed or uncorrelated noise and its rate per pixel, according to (6).

A primary event could be followed by avalanches due to correlated noise sources—AP and OCT. An AP event could cascade into a series of AP avalanches in the same pixel, while an OCT event could spatially cascade, triggering OCT avalanches in surrounding pixels. Furthermore, every AP event could trigger OCT avalanches in surrounding pixels and vice versa—a pixel triggered due to OCT could be triggered by AP afterwards. Thus, three coupled equations are required to derive the total avalanche rate of a single pixel in a SiPM.

$P_{xt}$, the probability that an OCT avalanche will be triggered in pixel $\left(i,j\right)$ due to an avalanche in one of its adjacent pixels, can be determined according to [11]. Hence, the rate of OCT-induced avalanches in the (i,j) pixel defined according to (7)

(A2) $$\lambda_{{xt}_{i,j}}=\left[\left(\sum_{\begin{array}{c} k=i-1\\ k\neq i \end{array}}^{i+1}\lambda_{{signal}_{k,j}}+{\lambda_{ap}}_{k,j}+\sum_{\begin{array}{c} l=j-1\\ l\neq j \end{array}}^{j+1}\lambda_{{signal}_{i,l}}+{\lambda_{ap}}_{i,l}\right)+4DCR\right]P_{xt}$$

The AP avalanche rate for the (i,j) pixel according to (8)

(A3) $$\lambda_{{ap}_{i,j}}=\left(\lambda_{{primary}_{i,j}}+\lambda_{{xt}_{i,j}}\right)\frac{P_{ap}}{1-P_{ap}}$$

The total avalanche rate of the (i,j) pixel according to (9)

(A4) $$\lambda_{{total}_{i,j}}=\lambda_{{primary}_{i,j}}+\lambda_{{ap}_{i,j}}+\lambda_{{xt}_{i,j}}$$

Equation (A2) describes $\lambda_{xt_{i,j}}$ as a linear combination of avalanche sources in neighboring pixels. As such, matrix formulation is applicable. Equation (A2) could be simplified using the definition of $\lambda_{primary}$

$$\lambda_{{xt}_{i,j}}=\left[\left(\sum_{\begin{array}{c} k=i-1\\ k\neq i \end{array}}^{i+1}\left(\lambda_{{primary}_{k,j}}-DCR+{\lambda_{ap}}_{k,j}\right)+\sum_{\begin{array}{c} l=j-1\\ l\neq j \end{array}}^{j+1}\left(\lambda_{{primary}_{i,l}}-DCR+{\lambda_{ap}}_{i,l}\right)\right)+4DCR\right]P_{xt}$$

The term for DCR cancels as the sums are over all pixels $\left(k,l\right)\neq (i,j)$. Hence, (A2) becomes

(A5) $$\lambda_{{xt}_{i,j}}=\left[\sum_{\begin{array}{c} k=i-1\\ k\neq i \end{array}}^{i+1}\left(\lambda_{{primary}_{k,j}}+{\lambda_{ap}}_{k,j}\right)+\sum_{\begin{array}{c} l=j-1\\ l\neq j \end{array}}^{j+1}\left(\lambda_{{primary}_{i,l}}+{\lambda_{ap}}_{i,l}\right)\right]P_{xt}$$

For convenience, define $\alpha =\frac{P_{ap}}{1-P_{ap}}$. Substituting (A3) in (A5) gives

$$\lambda_{{xt}_{i,j}}=\left[\sum_{\begin{array}{c} k=i-1\\ k\neq i \end{array}}^{i+1}\left(\lambda_{{primary}_{k,j}}+\alpha \left(\lambda_{{primary}_{k,j}}+\lambda_{{xt}_{k,j}}\right)\right)+\sum_{\begin{array}{c} l=j-1\\ l\neq j \end{array}}^{j+1}\left(\lambda_{{primary}_{i,l}}+\alpha \left(\lambda_{{primary}_{i,l}}+\lambda_{{xt}_{i,l}}\right)\right)\right]P_{xt}$$

(A6) $$\lambda_{{xt}_{i,j}}=\left[\sum_{\begin{array}{c} k=i-1\\ k\neq i \end{array}}^{i+1}\left(\left(\alpha +1\right)\lambda_{{primary}_{k,j}}+\alpha \lambda_{{xt}_{k,j}}\right)+\sum_{\begin{array}{c} l=j-1\\ l\neq j \end{array}}^{j+1}\left(\left(\alpha +1\right)\lambda_{{primary}_{i,l}}+\alpha \lambda_{{xt}_{i,l}}\right)\right]P_{xt}$$

To simplify, let us define column vectors of dimension N×1, where each element corresponds to the avalanche rate of a specific pixel in the array:

$$\lambda_{xt}=\left[\begin{array}{c} \lambda_{xt_{1}}\\ \vdots \\ \lambda_{xt_{N}} \end{array}\right];\lambda_{primary}=\left[\begin{array}{c} \lambda_{{primary}_{1}}\\ \vdots \\ \lambda_{{primary}_{N}} \end{array}\right]$$

The index m of a pixel located at position (i,j) within the array is determined by:

$$m=\left(i-1\right)n+j\;\;;\;\;1\leq m\leq N$$

Here, n denotes the number of pixels per row and $N=n^{2}$ is the total number of pixels in the array. This indexing scheme allows the two-dimensional pixel coordinates to be mapped into a one-dimensional vector representation, as seen in Figure 3.

To represent how pixel m might be triggered by OCT coming from avalanches in its nearest neighbors a coupling matrix, $A_{NxN}$, is defined as follows:

$$A_{q,r}=\left\{\begin{array}{c} 1,\\ 1,\\ \begin{array}{c} 1,\\ 1,\\ 0, \end{array} \end{array}\begin{array}{c} q=m,r=m+n\\ q=m,r=m-n\\ \begin{array}{c} q=m,r=m+1\\ \begin{array}{c} q=m,r=m-1\\ otherwise \end{array} \end{array} \end{array}\right.$$

where q represents the pixel triggered by OCT, and r represents the OCT source pixel. $A_{q,r}=1$ means pixel m could be triggered by OCT sourcing from its adjacent pixels, and $A_{q,r}=0$ means the pixel does not trigger an OCT to itself and non-adjacent pixels do not trigger OCT in pixel m as well.

Writing (A6) in matrix notation gives

(A7) $$\Lambda_{xt}=\left(\left(1+\alpha \right)A\Lambda_{primary}+\alpha A\Lambda_{xt}\right)P_{xt}$$

Rearranging (A7)

$$\Lambda_{xt}-P_{xt}\alpha A\Lambda_{xt}=P_{xt}\left(1+\alpha \right)A\Lambda_{primary}$$

$$\left(I-P_{xt}\alpha A\right)\Lambda_{xt}=P_{xt}\left(1+\alpha \right)A\Lambda_{primary}$$

(A8) $$\Lambda_{xt}={\left(I-P_{xt}\alpha A\right)}^{-1}AP_{xt}\left(1+\alpha \right)\Lambda_{primary}$$

Similarly, writing (A3) and (A4) in matrix notation gives

(A9) $$\Lambda_{ap}=\alpha \left(\Lambda_{primary}+\Lambda_{xt}\right)$$

(A10) $$\Lambda_{total}=\Lambda_{primary}+\Lambda_{ap}+\Lambda_{xt}$$

Substituting (A9) in (A10) gives

$$\Lambda_{total}=\Lambda_{primary}+\alpha \left(\Lambda_{primary}+\Lambda_{xt}\right)+\Lambda_{xt}$$

(A11) $$\Lambda_{total}=\left(1+\alpha \right)\left(\Lambda_{primary}+\Lambda_{xt}\right)$$

Substituting (A8) in (A11) and $\alpha =\frac{P_{ap}}{1-P_{ap}}$ gives a vector representing the total avalanche rate per pixel in the array

$$\Lambda_{total}=\left(1+\frac{P_{ap}}{1-P_{ap}}\right)\left(\Lambda_{primary}+P_{xt}\left(1+\frac{P_{ap}}{1-P_{ap}}\right){\left(I-P_{xt}\frac{P_{ap}}{1-P_{ap}}A\right)}^{-1}A\Lambda_{primary}\right)$$

(A12) $$\Lambda_{total}=\left(1+\frac{P_{ap}}{1-P_{ap}}\right)\left[I+P_{xt}\left(1+\frac{P_{ap}}{1-P_{ap}}\right){\left(I-P_{xt}\frac{P_{ap}}{1-P_{ap}}A\right)}^{-1}A\right]\Lambda_{primary}$$

Equation (A12) can be directly implemented in Equations (1) and (5) to get the detection rate of dSiPM (non-paralayzable SiPM) and aSiPM (paralyzable SiPM). Per pixel, the detection rate is

(A13) $$R_{{dSiPM}_{m}}=\min\left(\frac{{\Lambda_{total}}_{m}}{1+\Lambda_{{total}_{m}}t_{d}},\frac{1}{t_{d}}\right)$$

(A14) $$R_{{aSiPM}_{m}}=\min\left(\Lambda_{{total}_{m}}e^{-\Lambda_{{total}_{m}}t_{d}},\frac{1}{{et}_{d}}\right)$$

The total detection rate for each detector

(A15) $$R_{dSiPM}=\sum_{m=1}^{N}R_{{dSiPM}_{m}}$$

(A16) $$R_{aSiPM}=\sum_{m=1}^{N}R_{{aSiPM}_{m}}$$
